# Changes in patient functioning and disability: results from a phase 3, double-blind, randomized, placebo-controlled clinical trial evaluating galcanezumab for chronic migraine prevention (REGAIN)

**DOI:** 10.1007/s11136-020-02623-1

**Published:** 2020-09-15

**Authors:** Janet Ford, Cristina Tassorelli, Elizabeth Leroux, Shufang Wang, David Ayer, Russell Nichols, Holland Detke

**Affiliations:** 1 Lilly Research Laboratories, Lilly Corporate Center, Eli Lilly and Company, Indianapolis, IN 46285 USA; 2 IRCCS C. Mondino Foundation, Pavia, Italy; 3 Department of Brain and Behavioral Sciences, University of Pavia, Pavia, Italy; 4 Department of Clinical Neuroscience, University of Calgary, Calgary, Canada

**Keywords:** Galcanezumab, Calcitonin gene-related peptide, Chronic migraine, Disability, Patient-reported outcomes, Quality of life

## Abstract

**Purpose:**

To evaluate secondary outcomes including changes in functioning and disability associated with galcanezumab, a humanized monoclonal antibody to calcitonin gene-related peptide, in patients with chronic migraine.

**Methods:**

Patients randomly received galcanezumab (120 mg *n* = 278, 240 mg *n* = 277) or placebo (*n* = 558) during 3 months of double-blind treatment, followed by a 9-month open-label extension. The Migraine-Specific Quality-of-Life Questionnaire v2.1 (MSQv2.1) measured the impact of migraine on patient functioning. The Migraine Disability Assessment (MIDAS) quantified headache-related disability. Changes from baseline were analyzed with mixed model repeated measures or analysis of covariance.

**Results:**

Total MSQ score at baseline was 44.88 ± 18.02 (mean ± SD), indicating significant functional impairment. At Month 3, least squares (LS) mean change ± SE in total MSQ for galcanezumab-treated patients were 20.51 ± 1.49 (120 mg) and 20.49 ± 1.49 (240 mg), both statistically significantly greater vs placebo-treated patients (14.55 ± 1.21; both *P* < 0.001). Total MIDAS score at baseline was 67.24 ± 57.31 (mean ± SD). At Month 3, LS mean change ± SE from baseline in total MIDAS for galcanezumab-treated patients was statistically significantly greater than placebo for 120 mg group (placebo: − 11.53 ± 3.38 vs 120 mg: − 20.27 ± 4.07; *P* < 0.05) but not for 240 mg group (− 17.02 ± 4.05). At Month 12, within-group mean changes from baseline for total MSQ (28.56 ± 1.19 previous placebo; 29.53 ± 1.51 previous 120 mg; 25.83 ± 1.49 previous 240 mg) and MIDAS scores (− 28.47 ± 2.95 previous placebo; − 31.47 ± 3.69 previous 120 mg; − 31.13 ± 3.62 previous 240 mg) were statistically significant (*P* < 0.001) for the open-label treatment population regardless of previous double-blind treatment assignment.

**Conclusions:**

Galcanezumab-treated patients with chronic migraine reported statistically significant improvements in functioning and disability, representing a clinically significant change.

*****Trial registration***:**

ClinicalTrials.gov registry: NCT02614261. Registered 25 November 2015.

**Electronic supplementary material:**

The online version of this article (10.1007/s11136-020-02623-1) contains supplementary material, which is available to authorized users.

## Introduction

Migraine represents a spectrum of disorders consisting of episodic (i.e., < 15 headache days per month) and chronic forms (i.e., ≥ 15 headache days per month for ≥ 3 months) [1]. Population-based studies have indicated a rate of chronification from episodic migraine to chronic migraine of 2.5–3.0% annually [2], although rates as high as 14% have been reported in specialty headache clinics [3]. Risk factors associated with development of chronic migraine include high frequency of attacks (episodic migraine), overuse of acute migraine medications, ineffective acute therapy, obesity, anxiety and depression, and stressful life events [4–6]. Migraine may also present in its chronic form from the start [7]. While many clinical characteristics and sociodemographic factors of people with chronic migraine and episodic migraine are similar, the duration of headache (treated or untreated) is significantly longer with chronic migraine; a greater proportion of patients with chronic migraine experience severe pain and occupational disability [8, 9]

The prevalence of chronic migraine in the general population is lower than episodic migraine, with the 12-month prevalence of chronic migraine (International Classification of Headache Disorders-II criteria) ranging from 0.2 to 2.7% in studies across multiple countries and peaking during mid-life [10, 11]. Higher prevalence rates during prime working ages are concerning, particularly for people with chronic migraine who experience more disease burden than those with episodic migraine [9, 10]. Beyond pain, the array of negative consequences due to this debilitating neurological disease include increased global disability, difficulties at work, decreased vitality and fatigue, emotional problems, decreased mental health, decreased physical health, poor social functioning, and psychiatric comorbidities [12].

Reducing headache-related disability is recognized in guidelines across multiple regions as one of the primary goals for preventive drugs [13–15]. There are many preventive medications that have the potential to reduce migraine headache attack frequency and severity especially for episodic migraine [13]; however, few have been proven effective for the treatment of chronic migraine in robust clinical studies [6, 16]. In addition, the benefits of preventive treatment on health-related quality of life (HRQoL) and disability are often unmeasured [17]. Observational research suggests that the current level of impaired functioning and disability among the population with chronic migraine remains high, indicating a continuing unmet medical need for people with migraine [9].

Galcanezumab, a humanized monoclonal antibody that binds calcitonin gene-related peptide (CGRP), was recently approved for the prevention of migraine in adults based on its proven efficacy in reducing the number of monthly migraine headache days in patients with episodic [18, 19] and chronic migraine [20]. Based on the previously reported considerations, it is important to understand the implications to patient functioning in day-to-day activities (Migraine-Specific Quality of Life Questionnaire [MSQv2.1]) and changes in the levels of disability due to migraine (Migraine Disability Assessment [MIDAS]) among patients with chronic migraine when treated with galcanezumab. The research herein reports the performance of galcanezumab on the secondary outcomes of functioning and disability in a randomized, 3-month, double-blind, placebo-controlled, Phase 3 study (REGAIN) including the 9-month open-label extension period with two different dose-regimens of galcanezumab (120 and 240 mg). The study’s hypothesis is that galcanezumab significantly improves patient functioning and decreases patient disability.

## Methods

### Study design

Details of this study’s design have been described earlier [20]. Briefly, the study design consisted of five study periods: initial screening and washout (3–45 days); a prospective lead-in or baseline period for determining the frequency of migraine headache days or probable migraine headache (30–40 days); a double-blind treatment period (Month 1–3); a 9-month open-label extension period (Months 3–12); and a 4-month post-treatment (washout) period (Months 12–16). Patients were randomized to treatment with galcanezumab 120 mg (with a 240-mg loading dose), galcanezumab 240 mg, or placebo in a 1:1:2 ratio. Assignment to treatment was via computer-generated random sequence with an interactive web-response system. Randomization was stratified by country, acute headache medication overuse (yes/no) as determined during prospective baseline, and presence of concurrent migraine preventive (yes/no). Treatments were administered monthly during office visits by subcutaneous injection. During the open-label extension period, all patients received galcanezumab 240 mg beginning at Month 3, 120 mg at Month 4, then flexible dosing of galcanezumab 120 mg or 240 mg from Month 5 onward at the discretion of the investigator. Patients used an electronic handheld diary device to record their headache information, such as pain severity and duration, other related symptoms, and acute medication use. Diary entries were made every day during the baseline, treatment, and post-treatment periods; patients were permitted to take specified acute medications during these periods, as well.

The study was conducted at 116 headache and clinical research centers in 12 countries: Argentina, Canada, Czech Republic, Germany, Israel, Italy, Mexico, the Netherlands, Spain, Taiwan, the United Kingdom, and the United States. The first patient was enrolled in January 2016, and the last patient completed the double-blind portion of the study in March 2017. The study is registered at ClinicalTrials.gov (NCT02614261).

The study protocol was reviewed and approved by appropriate ethic review boards at each study site (see Supplementary Table 1) and was conducted according to Good Clinical Practice and the Declaration of Helsinki. Patients provided written informed consent before initiating study procedures.

### Patient selection

The patient population consisted of male and female patients aged 18–65 years, previously diagnosed with chronic migraine per the International Classification of Headache Disorder 3-beta [21] criteria (i.e., frequency of ≥ 15 headache days for more than 3 months with features of migraine on at least 8 days per month), and had migraine onset before 50 years of age. Patients were to have a history of at least 1 headache-free day per month for 3 months prior to entry, as well as at least 1 headache-free day during the prospective baseline period. Other preventive migraine medications were disallowed, except for stable doses of topiramate or propranolol in a limited number of patients (15%) [20].

### Outcome measures

The MSQv2.1 is a patient-reported HRQoL instrument developed to address functional limitations in day-to-day activities of specific concern to people with migraine [22, 23]. It assesses the impact of migraine on the physical, social, and emotional limitations over the past 4 weeks, spanning work or daily activities, relationships with family and friends, leisure time, productivity, concentration, energy, tiredness, and feelings [22, 23]. The instrument measures three domains that are scored independently: (1) Role Function-Restrictive (RF-R), (2) Role Function-Preventive (RF-P), and (3) Emotional Function (EF). The RF-R domain measures the degree to which migraine limits the performance of daily activities (items 1 through 7), the RF-P domain measures the degree to which migraine interrupts or stops the performance of day-to-day activities (items 8 through 11), and EF has three items addressing feelings of frustration and helplessness due to migraine (items 12 through 14) [22, 23]. The standard response options for each question included “none of the time,” “a little bit of the time,” “some of the time,” “a good bit of the time,” “most of the time,” and “all of the time” [22, 23]. The MSQv2.1 total raw score and each of the domain raw scores were transformed to ranges from 0 (worst functional health status) to 100 (best functional health status), with a positive change in scores reflecting improvements in daily functioning. As a result, transformed scores reflect the percentage of the total possible score [24, 25]. Previous research defined minimal important within-group differences from baseline for each domain (RF-R + 10.9; RF-P + 8.3; EF + 12.2) [26, 27]. The MSQv2.1 has been recommended by the National Institutes of Health as a core instrument for headache studies [28] with proven reliability, validity, and sensitivity to change in migraine [22, 23]. It is also a recommended outcome by the Guidelines for Clinical Trials of the International Headache Society [29]. As a result, it has been used in numerous clinical studies, including CGRP clinical trials [18, 19, 30, 31].

The MIDAS questionnaire quantifies headache-related disability [32]. This instrument has five items that capture the number of days of missed work, reduced productivity at work, missed household work, reduced productivity at home, and missed social events over the past 3 months. Each item is scored from 0 to 90. However, missed days are not double-counted as days with reduced productivity; therefore, the total score ranges from 0 to 270. Higher values indicate more disability due to headaches, and categorical grades for the severity of disability have been defined for the MIDAS [32–34]. Scores ranging from 0–5 indicate little or no disability (Grade I); 6–10 indicates mild disability (Grade II); 11–20 indicates moderate disability (Grade III); 21–40 indicates severe disability (Grade IV-A); and 41–270 indicates very severe disability (Grade IV-B) [9]. Treatment response has been defined in previous research as a reduction in the total score of a least 50% from baseline [27]. Changes from one grade to a lower grade level are also considered clinically meaningful, given the high correlation with clinical judgment on patients’ level of pain, degree of disability, and urgency for medical treatment [34]. The instrument is also considered highly reliable and valid [32, 33].

The MSQv2.1 was collected at baseline and then monthly during the double-blind and open-label extension periods; the MIDAS was collected at baseline, at Month 3 (end of double-blind period), and at Months 6, 9, and 12 during the open-label extension.

### Statistical analyses

For the primary publication [20], the primary objective tested the hypothesis that at least 1 dose of galcanezumab (120 or 240 mg/mo) was superior to placebo in the prevention of migraine in patients with chronic migraine as measured by the overall mean change from baseline in the number of monthly migraine headache days during the 3-month double-blind treatment period. Accordingly, the target sample size was 1140, based on the assumption of a 15% discontinuation rate and an effect size of 0.30 in the last month of the 3-month treatment phase, to provide approximately 95% power that at least 1 galcanezumab group would separate from placebo at a 1-sided 0.025 significance level.

In this secondary analysis, mean changes from baseline on the MSQv2.1 and MIDAS were analyzed for the double-blind treatment period, as well as for the double-blind and open-label treatment periods combined. Open-label results are presented by previous double-blind treatment (i.e., previous placebo, previous 120 mg, and previous 240 mg). For the MIDAS during the double-blind treatment period, analysis of covariance (ANCOVA) was used to analyze the change from baseline to Month 3. The ANCOVA model included treatment, country, baseline medication overuse, concurrent preventive treatment, and baseline MIDAS score as independent variables. For the MIDAS scale, during the double-blind and open-label treatment periods combined, and for the MSQv2.1, a mixed model repeated measures (MMRM) analysis was conducted for the change from baseline to each post-baseline measurement. MMRM analysis included treatment, country, month, baseline medication overuse, concurrent preventive treatment, treatment-by-month interaction, baseline value, and baseline-by-month interaction as model terms.

In addition, responder analyses were conducted for both the MSQv2.1 and MIDAS during the double-blind treatment period. MSQv2.1 responders were defined as achieving the minimal important difference threshold (RF-R + 10.9; RF-P + 8.3; EF + 12.2) [26, 27]. The MSQv2.1 responder indicators were analyzed with a pseudo-likelihood-based generalized linear mixed model repeated measures approach including treatment, baseline medication overuse, concurrent preventive treatment, month, treatment-by-month interaction, and corresponding baseline domain score as model terms. MIDAS responders were defined as those reaching a MIDAS total score response of at least 50% improvement from baseline. MIDAS responder indicator at Month 3 was analyzed using logistic regression with treatment, country, baseline medication overuse, concurrent preventive treatment, and baseline MIDAS total score as model terms.

All analyses were pre-specified and were two-sided assuming a significance level of 5%. All randomized and treated patients with baseline and at least one post-baseline data point were included. Statistical analyses were conducted using SAS Enterprise Guide 7.0, SAS Institute, Cary, NC.

## Results

### Patients

Baseline demographics and disease burden are shown in Table 1. A total of 1113 randomized patients received at least one dose of study drug and were included in the intent-to-treat (ITT) population (120 mg *N* = 278, 240 mg *N* = 277, placebo *N* = 558). Overall, 1037 patients (93.2% [1037/1113]) with chronic migraine completed treatment during the double-blind treatment period. A total of 1022 patients entered the open-label extension period, and 825 (80.7%) patients completed this period of the study.

**Table 1:** Baseline demographics, medical characteristics, and disease burden (intent-to-treat population)

	Placebo *N* = 558	Galcanezumab	
		120 mg *N* = 278	240 mg *N* = 277
Age, years, mean (SD)	41.63 (12.08)	39.66 (11.88)*	41.05 (12.40)
Gender (female), *n* (%)	483 (86.6)	237 (85.3)	226 (81.6)
Race (white), *n* (%)	432 (77.4)	223 (80.2)	224 (81.2)
Duration of migraine illness (years), mean (SD)	21.94 (12.85)	20.37 (12.74)	20.06 (12.72)*
Number of monthly migraine headache days, mean (SD)	19.55 (4.59)	19.36 (4.27)	19.17 (4.60)
Migraine headache days with acute medication use, mean (SD)	15.51 (6.57)	15.12 (6.25)	14.49 (6.25)*
Number of comorbidities, mean (SD)	4.39 (3.70)	4.08 (3.33)	4.21 (3.19)
MSQv2.1^a^	*N* = 546	*N* = 272	*N* = 272
Total, mean (SD)	44.38 (17.92)	45.19 (18.19)	45.58 (18.10)
RF-R, mean (SD)	38.37 (17.18)	39.29 (17.30)	38.93 (17.31)
RF-P, mean (SD)	55.01 (20.84)	55.48 (21.99)	57.13 (20.51)
EF, mean (SD)	44.22 (25.95)	45.27 (25.77)	45.71 (27.36)
MIDAS	*N* = 546	*N* = 272	*N* = 272
Total, mean (SD)	68.66 (57.36)	62.46 (49.48)	69.17 (64.08)
# of days missed work or school	4.97 (10.64)	4.45 (7.44)	5.71 (12.51)
# of days reduced productivity	15.55 (18.00)	14.02 (16.17)	15.94 (18.59)
# of days missed household work	18.39 (18.36)	17.29 (16.28)	18.65 (18.86)
# of days reduced productivity	17.79 (18.27)	16.25 (16.57)	17.86 (19.88)
# of days missed family/social	10.45 (13.84)	11.01 (15.61)	10.73 (14.74)

^#^number; *EF* emotional function; *MIDAS* migraine disability assessment; *MSQv2.1* migraine-specific quality of life questionnaire version 2.1; *RF-P* role function-preventive; *RF-R* role function-restrictive; *SD* standard deviation

^a^Total and each domain’s raw dimension scores were transformed to a 0–100 point scale

*p value comparison vs placebo *P* < 0.05

Mean number of monthly migraine headache days at baseline was 19.4 for the ITT population (Table 1). Previously reported overall mean reduction in the number of monthly migraine headache days at the end of the 3-month double-blind period was − 4.8 for 120 mg, − 4.6 for 240 mg, and − 2.7 for placebo (*P* < 0.001 for each dose compared to placebo) [20] and ranged from − 8.0 to − 9.0 at Month 12 for all patients who participated in the open-label extension study [35].

Mean baseline MSQv2.1 total scores and MIDAS scores did not differ significantly between galcanezumab groups and placebo (Table 1).

### Functional limitations: MSQ

**Fig. 1:**
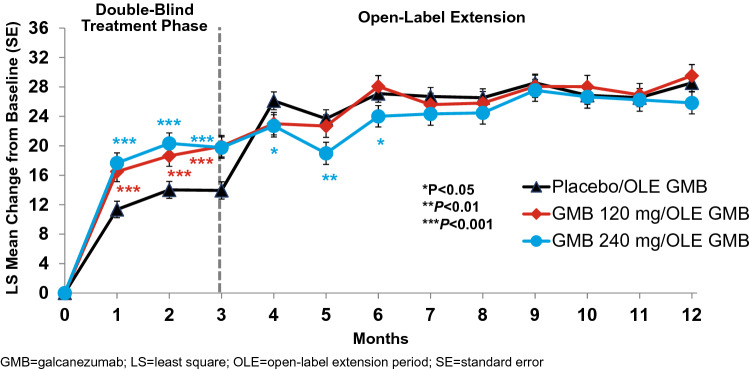
Least squares mean change from baseline ± standard error for Migraine-Specific Quality of Life total score

**Fig. 2:**
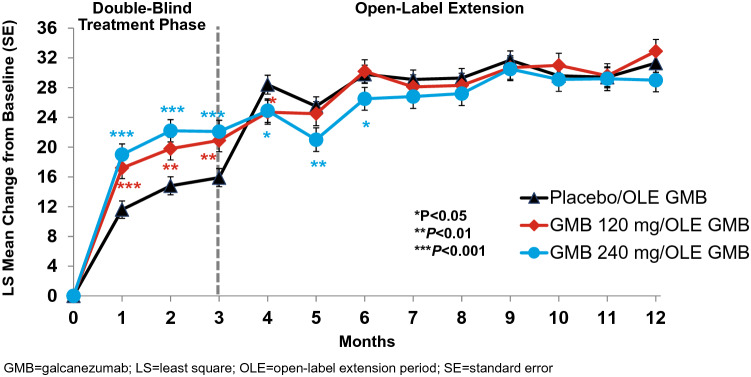
Least squares mean change from baseline ± standard error for Migraine-Specific Quality of Life Role Function Restrictive domain score

The MSQ total score at baseline was 44.88 ± 18.02 (mean ± SD), indicating a clinically significant functional impairment. Galcanezumab significantly increased daily functioning (*P* < 0.001) as measured by the total score and each domain of the MSQv2.1 in both the double-blind (Table 2) and open-label extension periods (Table 3). At Month 3, least squares (LS) mean change ± SE in the MSQ total scores for galcanezumab were 20.51 ± 1.49 (120 mg) and 20.49 ± 1.49 (240 mg), which corresponded to statistically significant greater improvements in functioning than placebo (14.55 ± 1.21; *P* < 0.001) (Table 2, Fig. 1). Both galcanezumab 120 mg and 240 mg showed significant increases in the LS means (*P* < 0.001) and difference vs placebo (*P* < 0.001) of the MSQ total score and RF-R, RF-P, and EF domains at Month 3 (Table 2). Moreover, the greater improvement in total score and RF-R domain score in the galcanezumab treatment groups compared to placebo was already evident at Month 1 (each *P* < 0.001) (Fig. 2). The improvement in daily functioning was also apparent at Month 4 in the group that switched from placebo to galcanezumab and was sustained monthly through Month 12 during the open-label extension period in all three previous treatment groups. At Month 12, within-group changes from baseline for the MSQ total score reflected statistically significant improvements in patient functioning (*P* < 0.001) for each previous treatment group (Table 3). At the end of the open-label extension period (Month 12), patients’ average level of functional impairment for the pooled galcanezumab population was reduced from greater than 50% to less than 30%.

**Table 2:** Changes in patient functioning and disability scores during the 3-month double-blind treatment period

		Galcanezumab	
		120 mg	240 mg
MSQv2.1 total	*N* = 494	*N* = 252	*N* = 253
LS mean change (SE)	14.55 (1.21)**	20.51 (1.49)**	20.49 (1.49)**
Diff vs placebo (SE)	–	5.96 (1.51)^a^	5.93 (1.51)^a^
95% CI	–	3.0, 8.9	3.0, 8.9
RF-R			
LS mean change (SE)	16.76 (1.18)**	21.81 (1.41)**	23.05 (1.63)**
Diff vs placebo (SE)	–	5.06 (1.50)^a^	6.29 (1.66)^a^
95% CI	–	2.1, 8.0	3.0, 9.6
RF-P			
LS mean change (SE)	10.98 (1.15)**	17.98 (1.42)**	16.07 (1.41)**
Diff vs placebo (SE)	–	7.00 (1.44)^a^	5.09 (1.44)^a^
95% CI	–	4.2, 9.8	2.3, 7.9
EF			
LS mean change (SE)	14.07 (1.55)**	21.03 (1.91)**	20.70 (1.90)**
Diff vs placebo (SE)	–	6.96 (1.94)^a^	6.62 (1.93)^a^
95% CI	–	3.2, 10.8	2.8, 10.4
MIDAS total	*N* = 504	*N* = 254	*N* = 258
LS mean change (SE)	− 11.53 (3.38)	− 20.27 (4.07)	− 17.02 (4.05)
Diff vs placebo (SE)	–	− 8.74 (3.90)^b^	− 5.49 (3.88)
95% CI	–	− 16.4, − 1.1	− 13.1, 2.1
MIDAS number of days missed work or school			
LS mean change (SE)	− 0.36 (0.72)	− 1.86 (0.87)	− 0.29 (0.86)
Diff vs placebo (SE)	–	− 1.50 (0.83)	0.07 (0.82)
95% CI	–	− 3.1, 0.1	− 1.5, 1.7
MIDAS number of days reduced productivity at work or school			
LS mean change (SE)	− 2.62 (1.01)	− 3.87 (1.22)	− 3.49 (1.21)
Diff vs placebo (SE)	–	− 1.25 (1.17)	− 0.87 (1.16)
95% CI	–	− 3.5, 1.0	− 3.2, 1.4
MIDAS number of days missed household work			
LS mean change (SE)	− 3.51 (0.95)	− 6.09 (1.15)	− 5.80 (1.14)
Diff vs placebo (SE)	–	− 2.58 (1.10)^b^	− 2.29 (1.09)^b^
95% CI	–	− 4.7, − 0.4	− 4.4, − 0.2
MIDAS number of days reduced productivity in household work			
LS mean change (SE)	− 3.35 (0.94)	− 5.59 (1.13)	− 4.43 (1.12)
Diff vs placebo (SE)	–	− 2.24 (1.08)^b^	− 1.08 (1.07)
95% CI	–	− 4.4, − 0.1	− 3.2, 1.0
MIDAS number of days missed family/social			
LS mean change (SE)	− 1.37 (0.83)	− 3.20 (1.00)	− 2.46 (1.00)
Diff vs placebo (SE)	–	− 1.83 (0.96)	− 1.09 (0.95)
95% CI	–	− 3.7, − 0.1	− 3.0, 0.8

*CI* confidence interval; *Diff* difference; *EF* emotional function; *LS* least squares; *MIDAS* migraine disability assessment; *MSQv2.1* migraine-specific quality of life questionnaire v2.1; *RF-P* role function-preventive; *RF-R* role function-restrictive; *SE* standard error

^a^*P*  <0.001 vs placebo

^b^*P*  <0.05 vs placebo

**P < 0.001 vs baseline

**Table 3:** Least square mean changes from baseline in patient functioning and disability scores during open-label treatment period (Month 12)

	Galcanezumab		
	Previous Placebo	Previous 120 mg	Previous 240 mg
MSQv2.1	N = 399	N = 193	N = 199
Total	28.56 (1.19)**	29.53 (1.51)**	25.83 (1.49)**
RF-R	31.34 (1.24)**	32.91 (1.58)**	28.98 (1.56)**
RF-P	23.32 (1.12)**	23.97 (1.43)**	20.24 (1.41)**
EF	28.87 (1.49)**	29.27 (1.89)**	26.17 (1.87)**
MIDAS	N = 396	N = 193	N = 199
Total	− 28.47 (2.95)**	− 31.47 (3.69)**	− 31.13 (3.62)**
MIDAS number of days missed work or school	− 1.26 (0.59)*	− 2.73 (0.74)**	− 2.08 (0.72)*
MIDAS number of days reduced productivity at work or school	− 7.03 (0.82)**	− 6.83 (1.03)**	− 6.68 (1.01)**
MIDAS number of days missed household work	− 8.17 (0.79)**	− 8.74 (0.99)**	− 8.89 (0.98)**
MIDAS number of days reduced productivity in household work	− 7.97 (0.77)**	− 8.43 (0.97)**	− 8.47 (0.95)**
MIDAS number of days missed family/social	− 4.31 (0.68)**	− 5.37 (0.86)**	− 5.13 (0.84)**

*CI* confidence interval; *Diff* difference; *EF* emotional function; *LS* least squares; *MIDAS* migraine disability assessment; *MSQv2.1* migraine-specific quality of life questionnaire v2.1; *RF-P* role function-preventive; *RF-R* role function-restrictive; *SE* standard error

All *P* values are within-group comparisons vs baseline: *P < 0.05; **P < 0.001

Galcanezumab treatment produced a greater proportion of patients who met the established minimal important difference criteria for improving patient functioning at Month 3 for MSQv2.1 domains compared to treatment with placebo. For each domain, the estimated percentage of patients in each treatment group that met the criteria ± SE and level of significance for the 120 and 240 mg treatment groups vs placebo, respectively, are RF-R: 77.6% ± 2.8 (*P* < 0.001), 75.2% ± 2.9 (*P* = 0.002), and 63.6% ± 2.6; RF-P: 76.1% ± 2.9 (*P* < 0.001), 73.1% ± 3.0 (*P* < 0.001), and 58.0% ± 2.7; EF: 67.8% ± 3.3 (*P* = 0.003), 68.3% ± 3.2 (*P* = 0.002), and 56.1% ± 2.7.

### Disability: MIDAS

The MIDAS mean ± SD score at baseline was 67.24 ± 57.31, indicating very severe disability (Table 1). At Month 3, the difference in the LS mean change ± SE from baseline in the MIDAS total score for galcanezumab indicated a decrease in disability that was significantly greater for the 120 mg dose only (− 8.74 ± 3.90; *P* < 0.05) and similar for the 240 mg dose (− 5.49 ± 3.88) compared with placebo (− 11.53 ± 3.38) (Table 2). The percentage of patients meeting the definition of ≥ 50% response at Month 3 (model estimated rate) was significantly greater for both the galcanezumab 120 mg and 240 mg treatment groups (48.8% and 45.0%, respectively; *P* < 0.02) vs placebo (35.8%).

**Fig. 3:**
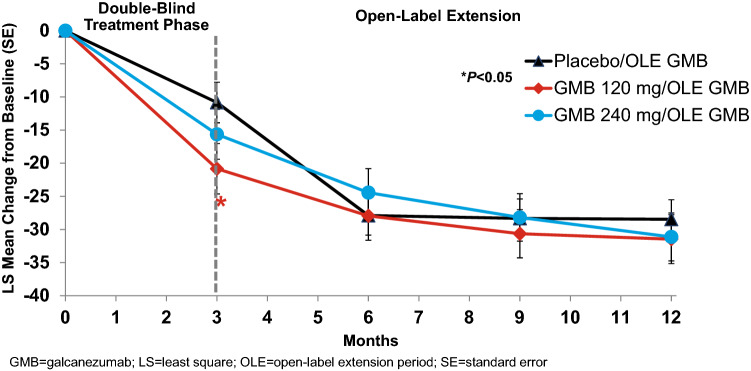
Least squares mean change from baseline ± standard error for MIDAS total score

At the end of the double-blind treatment period (Month 3), several individual item scores of the MIDAS were significantly reduced with galcanezumab treatment vs placebo including fewer number of days of missed household work and reduced productivity in household work for the 120 mg group (*P* < 0.05) and number of days of missed household work for the 240 mg group (*P* < 0.05). At Month 12, within-group changes from baseline for the MIDAS total score reflected statistically significant (*P* < 0.001) reductions in disability across all three groups (Table 3). Specifically, disability was reduced from Grade level IV-B to Grade level IV-A for the pooled galcanezumab group, the average decrease in the MIDAS total score was greater than 30 points (− 31.47 for 120 mg, − 31.13 for 240 mg) compared with baseline (62.46 for 120 mg, 69.17 for 240 mg). At Month 12, within-group changes from baseline for the five individual item scores indicated reductions in disability that were statistically significant (*P* < 0.05) for each previous treatment group. The reductions in MIDAS total scores persisted during the open-label extension period and were similar across all previous treatment groups (Fig. 3).

## Discussion

The physical and emotional impact of migraine on daily activities, as assessed by the MSQv2.1, indicated a high degree of functional impairment in this chronic migraine population, given baseline scores less than 50 out of 100 total possible points (100 indicates no functional impairment due to migraine). When compared to placebo after only 3 months of treatment with galcanezumab, statistically significant and clinically meaningful improvements in daily functioning as measured by the MSQv2.1 were observed, with significant reductions occurring at Month 1 for the total score and each of the three domains. Improvements with galcanezumab were considered clinically meaningful as demonstrated by the published analyses results for the established minimal important difference thresholds [26, 27]; a significantly greater proportion of patients on galcanezumab reached this degree of improvement when compared to placebo. During the open-label extension period, improvements in patient functioning were sustained. Reductions in disability were also statistically significantly greater for the 120 mg galcanezumab group when compared to placebo at Month 3 as measured by the MIDAS. The decrease in the level of disability was considered clinically meaningful given that a significantly greater proportion of patients on galcanezumab met the previously published responder threshold of greater than a 50% reduction when compared to placebo [27]. Notable improvements were observed in patient functioning, with impairment levels less than 30% at the end of 12 months, and disability was reduced from Grade level IV-B to Grade level IV-A.

The baseline demographics, disease characteristics, and levels of functioning and disability for this clinical trial population were consistent with the general chronic migraine population and are reflective of a debilitating neurological disease [9, 36]. The baseline characteristics of this population included more than 19 migraine headache days/month, duration of illness of approximately 21 years, and on average more than four comorbid conditions. Measures of disability and functional impairment indicated severe illness due to migraine in this population [9, 37]. Accordingly, this is a very ill population who should be at their prime productivity but instead are missing work/school, home responsibilities, and social events, which greatly impacts society. Reducing burden of migraine (e.g., headache-related disability) in patients with chronic migraine is a primary treatment goal per preventive treatment guidelines [13–15]. The present findings show that galcanezumab positively affects functional limitations and disability in adult patients with chronic migraine.

The findings described herein with galcanezumab as a preventive medication for patients with chronic migraine parallel and extend the findings of other relatively new preventive drugs [26, 27]. In a randomized, 16-week, double-blind, placebo-controlled study of 306 topiramate-treated patients with chronic migraine, daily functioning as measured by the MSQv2.1 was significantly improved at Week 4 in all three domains and at Weeks 8 and 16 in both RF-P and EF domains (*P* < 0.05) [27]. Although not statistically significant, 56% of topiramate-treated patients (100 mg/day) vs 45% of placebo-treated patients reported > 50% improvement from baseline in MIDAS scores (*P* = 0.07); the lack of significance was attributed to missing data, topiramate patients having higher disability at baseline, and overlapping recall periods. Data from the randomized, double-blind, placebo-controlled PREEMPT trial that included 1384 patients with chronic migraine found that onabotulinumtoxinA (155–195 U every 12 weeks) was associated with significant and clinically meaningful improvements in all domains of the MSQv2.1 at Weeks 12 and 24 (*P* < 0.001) [26]. At Week 12, MSQ mean changes from baseline in RF-R, RF-P, and EF scores following onabotulinumtoxinA were 16.2, 13.0, and 18.3, respectively [26]. Migraine-specific HRQoL and disability, as measured by the MSQv2.1 and MIDAS, were statistically improved in patients with chronic migraine receiving erenumab (70 mg or 140 mg) vs placebo in a Phase 2, randomized, double-blind study [38]. For example, at Week 12, mean changes for placebo vs the 70 mg and 140 mg groups were 11.8 vs 17.7 (*P* = 0.002) and 19.1 (*P* < 0.001), respectively, for RF-R scores. Lipton et al. also reported that 12-week preventive treatment with fremanezumab was associated with significantly improved RF-R and RF-P MSQv2.1 domain scores from baseline compared to placebo in patients with chronic migraine [39].

The research presented in this paper must be interpreted relative to known strengths and limitations. Whether the findings presented herein can be applied to the broader general population of patients with chronic migraine is not known. However, the baseline characteristics of our patients were similar to patients with chronic migraine described elsewhere [9, 36]. In this study, HRQoL outcomes were reported in patients with chronic migraine who received galcanezumab; only a limited number of patients used concurrent topiramate or propranolol (15%); and changes in HRQoL outcomes for this specific subgroup were not evaluated due to the small number. Placebo response was observed during the double-blind treatment period for the HRQoL outcomes, which is not unexpected given that patient experiences and expectations for pain treatments are known confounders [40]. Interestingly, analyses of subgroups with a history of migraine preventive treatment failures revealed lower placebo response rates, providing further evidence of the complex relationship between treatment expectations and placebo response in patients with migraine [41]. Additional research is needed for a more complete understanding of the complex factors involved in the placebo response among patients with migraine.

Inclusion of the 9-month open-label extension analysis provides added confidence in longer-term findings; however, this may not be reflective of disease management in real-world clinical practice. Accordingly, there are challenges with interpretation of the HRQoL findings due to patient experience and expectations. The MSQv2.1 and MIDAS measure two different constructs, that is, migraine-specific functional impairment and disability; however, the MSQv2.1 seemed more sensitive to measuring changes in this chronic migraine population, possibly due to the inclusiveness of emotional implications. The MIDAS may have measurement bias associated with employment status and engagement with household activities [32, 33]. Although improvement in disability for the 240 mg galcanezumab group was not significantly different compared to placebo at Month 3, the direction of the score change suggested more improvement, and there may have been insufficient power to detect a statistically significant difference with the MIDAS for this secondary outcome. Also, the recall period for the two measures differed; however, results and conclusions were directionally similar for these two HRQoL instruments.

## Conclusions

Given the debilitating nature of chronic migraine, clinicians and patients need information on multiple health-related outcomes when evaluating preventive treatments. Clinically meaningful reductions in daily functional impairment and disability due to migraine translates into significant societal benefits associated with work productivity, performance at home, and social health. The findings of this study provide important evidence for clinicians and health care policy makers, that beyond improvement in the number of migraine headache days, galcanezumab increased patients’ HRQoL scores and decreased disability scores in a rapid and sustained fashion.

## Electronic supplementary material

Below is the link to the electronic supplementary material.

Supplementary file 1 (DOCX 17 kb)

## Data Availability

Lilly provides access to all individual participant data collected during the trial, after anonymization, with the exception of pharmacokinetic or genetic data. Data are available to request 6 months after the indication studied has been approved in the US and EU and after primary publication acceptance, whichever is later. No expiration date of data requests is currently set once data are made available. Access is provided after a proposal has been approved by an independent review committee identified for this purpose and after receipt of a signed data sharing agreement. Data and documents, including the study protocol, statistical analysis plan, clinical study report, blank or annotated case report forms, will be provided in a secure data sharing environment. For details on submitting a request, see the instructions provided at www.vivli.org

## Abbreviations

ANCOVA: Analysis of covariance
CGRP: Calcitonin gene-related peptide
EF: Emotional function
HRQoL: Health-related quality of life
LS: Least squares
MIDAS: Migraine Disability Assessment
MMRM: Mixed model repeated measures
MSQ: Migraine-Specific Quality-of-Life Questionnaire v2.1
RF-P: Role function-preventive
RF-R: Role function-restrictive
SE: Standard error

## References

[1] Aurora SK, Brin MF (2017). Chronic migraine: An update on physiology, imaging, and mechanism of action of two available pharmacologic therapies. Headache.

[2] Bigal ME, Serrano D, Buse D, Scher A, Stewart WF, Lipton RB (2008). Acute migraine medications and evolution from episodic to chronic migraine: A longitudinal population-based study. Headache.

[3] Katsarava Z, Schneeweiss S, Kurth T, Kroener U, Fritsche G, Eikermann A (2004). Incidence and predictors for chronicity of headache in patients with episodic migraine. Neurology.

[4] Schwedt TJ (2014). Chronic migraine. BMJ.

[5] May A, Schulte LH (2016). Chronic migraine: Risk factors, mechanisms and treatment. Nature Reviews Neurology.

[6] Lipton RB, Silberstein SD (2015). Episodic and chronic migraine headache: Breaking down barriers to optimal treatment and prevention. Headache.

[7] Mathew NT, Reuveni U, Perez F (1987). Transformed or evolutive migraine. Headache.

[8] Katsarava Z, Buse DC, Manack AN, Lipton RB (2012). Defining the differences between episodic migraine and chronic migraine. Current Pain and Headache Reports.

[9] Blumenfeld AM, Varon SF, Wilcox TK, Buse DC, Kawata AK, Manack A (2011). Disability, HRQoL and resource use among chronic and episodic migraineurs: Results from the International Burden of Migraine Study (IBMS). Cephalalgia.

[10] Buse DC, Manack AN, Fanning KM, Serrano D, Reed ML, Turkel CC (2012). Chronic migraine prevalence, disability, and sociodemographic factors: results from the American Migraine Prevalence and Prevention Study. Headache.

[11] Steiner TJ, Stovner LJ, Katsarava Z, Lainez JM, Lampl C, Lantéri-Minet M (2014). The impact of headache in Europe: Principal results of the Eurolight project. The Journal of Headache and Pain.

[12] Raggi A, Giovannetti AM, Quintas R, D'Amico D, Cieza A, Sabariego C (2012). A systematic review of the psychosocial difficulties relevant to patients with migraine. The Journal of Headache and Pain.

[13] Silberstein SD, Holland S, Freitag F, Dodick DW, Argoff C, Ashman E (2012). Quality Standards Subcommittee of the American Academy of Neurology and the American Headache Society. Evidence-based guideline update: pharmacologic treatment for episodic migraine prevention in adults: Report of the Quality Standards Subcommittee of the American Academy of Neurology and the American Headache Society. Neurology.

[14] Pringsheim T, Davenport W, Mackie G, Worthington I, Aube M, Christie SN, Canadian Headache Society Prophylactic Guidelines Development Group (2012). Canadian Headache Society guideline for migraine prophylaxis. Canadian Journal of Neurological Sciences.

[15] Evers S, Afra J, Frese A, Goadsby PJ, Linde M, May A, EFNS (2009). EFNS guideline on the drug treatment of migraine—revised report of an EFNS task force. European Journal of Neurology.

[16] Diener H-C, Solbach K, Holle D, Gaul C (2015). Integrated care for chronic migraine patients: Epidemiology, burden, diagnosis and treatment options. Journal of Clinical Medicine.

[17] Abu Bakar N, Tanprawate S, Lambru G, Torkamani M, Jahanshahi M, Matharu M (2016). Quality of life in primary headache disorders: A review. Cephalalgia.

[18] Stauffer VL, Dodick DW, Zhang Q, Carter JN, Ailani J, Conley RR (2018). Evaluation of galcanezumab for the prevention of episodic migraine: The EVOLVE-1 randomized clinical trial. JAMA Neurology.

[19] Skljarevski V, Matharu M, Millen BA, Ossipov MH, Kim BK, Yang JY (2018). Efficacy and safety of galcanezumab for the prevention of episodic migraine: Results of the EVOLVE-2 phase 3 randomized controlled clinical trial. Cephalalgia.

[20] Detke HC, Goadsby PJ, Wang S, Friedman DI, Selzler KJ, Aurora SK (2018). Galcanezumab in chronic migraine: The randomized, double-blind, placebo-controlled REGAIN study. Neurology.

[21] Headache Classification Committee of the International Headache Society (IHS) (2013). The international classification of headache disorders, 3rd edition (beta version). Cephalalgia.

[22] Cole JC, Lin P, Rupnow MFT (2007). Validation of the Migraine-Specific Quality of Life Questionnaire version 2.1 (MSQ v. 2.1) for patients undergoing prophylactic migraine treatment. Quality of Life Research.

[23] Rendas-Baum R, Bloudek LM, Maglinte GA, Varon SF (2013). The psychometric properties of the Migraine-specific Quality of Life Questionnaire version 2.1 (MSQ) in chronic migraine patients. Quality of Life Research.

[24] Jhingran P, Davis SM, Lavange LM, Miller DW, Helms RW (1998). MSQ: Migraine-specific Quality-of-Life Questionnaire. Further investigation of the factor structure. Pharmacoeconomics.

[25] Jhingran P, Osterhaus JT, Miller DW, Lee JT, Kirchdoerfer L (1998). Development and validation of the Migraine-specific Quality of Life Questionnaire. Headache.

[26] Lipton RB, Varon SF, Grosberg B, McAllister PJ, Freitag F, Aurora SK (2011). OnabotulinumtoxinA improves quality of life and reduces impact of chronic migraine. Neurology.

[27] Dodick DW, Silberstein S, Saper J, Freitag FG, Cady RK, Rapoport AM (2007). The impact of topiramate on health-related quality of life indicators in chronic migraine. Headache.

[28] National Institute of Neurological Disorders and Stroke (NINDS). (2017). Summary of core and supplemental—highly recommended recommendations: headache. CDEs. https://deltasigmastats.com/sites/nindscde/files/Doc/Headache/CDEStartupResource_Headache.pdf. Accessed August 08, 2018.

[29] Tassorelli C, Diener HC, Dodick DW, Silberstein SD, Lipton RB, Ashina M, International Headache Society Clinical Trials Standing Committee (2018). Guidelines of the International Headache Society for controlled trials of preventive treatment of chronic migraine in adults. Cephalalgia.

[30] Dodick DW, Silberstein SD, Bigal ME, Yeung PP, Goadsby PJ, Blankenbiller T (2018). Effect of fremanezumab compared with placebo for prevention of episodic migraine: A randomized clinical trial. JAMA.

[31] Buse DC, Lipton RB, Hallström Y, Reuter U, Tepper SJ, Zhang F (2018). Migraine-related disability, impact, and health-related quality of life among patients with episodic migraine receiving preventive treatment with erenumab. Cephalalgia.

[32] Stewart WF, Lipton RB, Kolodner K, Liberman J, Sawyer J (1999). Reliability of the migraine disability assessment score in a population-based sample of headache sufferers. Cephalalgia.

[33] Stewart WF, Lipton RB, Dowson AJ, Sawyer J (2001). Development and testing of the Migraine Disability Assessment (MIDAS) questionnaire to assess headache-related disability. Neurology.

[34] Lipton RB, Stewart WF, Sawyer J, Edmeads JG (2001). Clinical utility of an instrument assessing migraine disability: The Migraine Disability Assessment (MIDAS) questionnaire. Headache.

[35] Detke HC, Li LQ, Wang S, Aurora SK (2018). One-year treatment with galcanezumab in patients with chronic migraine: Results from the open-label phase of the REGAIN study. Cephalalgia.

[36] Payne KA, Varon SF, Kawata AK, Yeomans K, Wilcox TK, Manack A (2011). The International Burden of Migraine Study (IBMS): Study design, methodology, and baseline cohort characteristics. Cephalalgia.

[37] Bagley CL, Rendas-Baum R, Maglinte GA, Yang M, Varon SF, Lee J (2012). Validating Migraine-Specific Quality of Life Questionnaire v2.1 in episodic and chronic migraine. Headache.

[38] Tepper S, Lipton R, Reuter U, Silberstein S, Stewart W, Leonardi D (2017). Patient-reported outcomes in patients with chronic migraine receiving placebo or erenumab (AMG) 334) in a phase 2, randomized, double-blind study. Neurology.

[39] Lipton, R. B., Fitzgerald, T., Cohen, J. M., Gandhi, S. K. (2018). The impact of fremanezumab on migraine-specific health-related quality of life in chronic migraine patients with concomitant preventive medication use. The 17^th^ Biennial Migraine Trust International Symposium, London, England, 06–09 September 2018. MTIS2018–114.

[40] Vase L, Amanzio M, Price DD (2015). Nocebo vs. placebo: The challenges of trial design in analgesia research. Clinical Pharmacology & Therapeutics.

[41] Ruff DD, Ford JH, Tockhorn-Heidenreich A, Sexson M, Govindan S, Pearlman EM (2019). Efficacy of galcanezumab in patients with chronic migraine and a history of preventive treatment failure. Cephalalgia.

