# Supplementary material for: Changes in patient functioning and disability: results from a phase 3, double-blind, randomized, placebo-controlled clinical trial evaluating galcanezumab for chronic migraine prevention (REGAIN)
Source: Qual Life Res. 2020 Sep 15;30(1):105–15. doi: 10.1007/s11136-020-02623-1 (PMC7847867; doi:10.1007/s11136-020-02623-1)
Supplement: Supplementary file 1 — (DOCX 17 kb) [file 11136_2020_2623_MOESM1_ESM.docx]

Supplementary Table 1

| Ethics Review Boards |
| --- |
| Quorum Review, Inc.  1501 Fourth Avenue Suite 800  Seattle, WA, 98101,  United States |
| Dean Foundation for Health Research and Education  2711 Allen Blvd., Suite 300 Middleton, WI 53562  United States |
| Office of Research Compliance  1600 Hampton Street, Suite 414 Columbia, SC 29208  United States |
| Baylor, Scott, & White  3310 Live Oak St.  Dallas, TX 75204  United States |
| IRB Services  372 Hollandview Trail Suite 300  Aurora, Ontario, L4G0A5,  Canada |
| Montreal Neurological Institute and Hospital  3801 Rue University, NEU REB Room 686 Montreal, Quebec, H3A 2B4  Canada |
| West Midlands - Edgbaston REC  The Old Chapel Royal Standard Place Nottingham, NG1 6FS  United Kingdom |
| Isala Klinieken METC  Gebouw M (Mondriaan), kamer 0.25, Postbus 10400 Dokter van Deenweg 1 Zwolle, 8025 BP  Netherlands |
| Hospital Universitari Vall d'Hebron  Passeig Vall d'Hebron, 119-129 Comité Ético de Investigación Clínica Barcelona, Barcelona, 08035  Spain |
| Comitato Etico Irccs San Raffaele Pisana  Via Val Cannuta 247 ROMA, 00166  Italy |
| Comitato Etico Interaziendale Bologna-IMOLA  Via Castiglione, 29 Bologna, Bologna, 40100  Italy |
| Comitato Etico Area Vasta Centro Presso AOU Careggi  L.go Brambilla 3 Firenze, Firenze, 50134  Italy |
| Comitato Etico della Provincia di Modena  Via Largo del Pozzo 71 Modena, Modena, 41124  Italy |
| Comitato Etico Ospedale San Raffaele  Via Olgettina, 60 Milano, 20132  Italy |
| Eticka komise IKEM a Thomayerovy nemocnice  Videnska 800, Multicentricka eticka komise Praha 4 – Krc, 140 59  Czech Republic |
| Eticka komise Clintrial, s.r.o.  Pocernicka 1427/16 Praha 10, 100 00  Czech Republic |
| Eticka komise FN u sv. Anny v Brne  Pekarska 53 Brno, 65691  Czech Republic |
| Ethikkommission der Landesärztekammer Hessen  Im Vogelsgesang 3 Frankfurt am Main, Hessen, 60488  Germany |
| Comite de Etica del Centro de Osteopatias Medicas  Azcuenaga 1860, 8° Floor CABA, Buenos Aires, C1128AAF  Argentina |
| Sanatorio Allende-Cordoba  Av. Hipolito Yrigoyen 384 Cordoba, Cordoba, X5000JHGQ  Argentina |
| Comite de Etica Independiente en Invest.Clinica Dr C Barclay  Larrea 1381 3°"A" Ciudad Autonoma de Buenos Air, C1117ABK  Argentina |
| Instituto Reumatologico Strusberg  Av. Emilio Olmos 247  Cordoba, Cordoba, X5000EDC  Argentina |
| Hillel Yaffe Medical Center  ERB, P. O. Box 169  Hadera, 38100  Israel |
| Chaim Sheba Medical Center  2 Sheba Road Tel Hashomer  Ramat Gan, 5266202  Israel |
| Maccabi Healthcare Services, Kfar Saba  ERB at Assuta Medical Center, Habarzel 20 St., Tel Aviv  4420122  Israel |
| Tel Aviv Sourasky Medical Center  6 Weizman St  Tel-Aviv Jaffa, 6423906  Israel |
| Chi-Mei Medical Center - Yung Kang  Institutional Review Board  IRB office, 4F Medical Building III, No. 901, Chung Hwa Rd. Yung-Kang Dist. Tainan, 71004  Taiwan |
| Kaohsiung Medical University Chung-Ho Memorial Hospital  IRB office, 8F, A Building, No. 100, Tzyou 1st Road Kaohsiung 80756  Taiwan |
| Sin-Lau Hospital  No. 57, Sec. 1, Dongmen Rd. Tainan, 70142  Taiwan |
| Taipei Veterans General Hospital  Institutional Review Board  No. 201 Shih-Pai Road Sec. 2, Taipei, 112  Taiwan |
| Far Eastern Memorial Hospital Research Ethics Review Committee  B1 South Building, No. 21, Sec. 2, Nanya S. Road, Banciao Dist New Taipei City, 220  Taiwan |
| Grupo Médico CAMINO S.C.  Av. Cuauhtemoc 1251, Col. Santa Cruz Atoyac Mexico City, Mexico City, 03310  Mexico |
| Hospital Angeles de Culiacan  Blvd. Alfonso G. Calderon Belarde #2193-A Interior 802, Desarrollo Urbano Tres Rios Culiacan, Sinaloa, 80020  Mexico |
| Estimulación Magnetica Transcraneal de Mexico  Sierra Candela 111-306 Lomas de Chapultepec Mexico City, Distrito Federal, 11000  Mexico |
